# Quantitative Deviation of Nanocrystals Using the RIR Method in X-ray Diffraction (XRD)

**DOI:** 10.3390/nano12142320

**Published:** 2022-07-06

**Authors:** Qinyuan Huang, Chunjian Wang, Quan Shan

**Affiliations:** 1 Faculty of Material Science and Engineering, Kunming University of Science and Technology, Kunming 650093, China; huangqinyuan@stu.kust.edu.cn; 2 National & Local Joint Engineering Laboratory of Advanced Metal Solidification Forming and Equipment Technology, Kunming University of Science and Technology, Kunming 650093, China; 3 Research Center for Analysis and Measurement, Kunming University of Science and Technology, Kunming 650093, China; 4 Analytic & Testing Research Center of Yunnan, Kunming 650093, China

**Keywords:** X-ray diffraction (XRD), quantitative phase analysis (QPA), reference intensity ratio (RIR), instrumental broadening, nanocrystal quantification

## Abstract

The reference intensity ratio (RIR) method, using X-ray diffraction (XRD), is considered one most of the rapid and convenient approaches for phase quantification in multi-phase mixture, in which nanocrystals are commonly contained in a mixture and cause a broadening of the diffraction peak, while another broadening factor, instrumental broadening, does not attract enough attention in related quantitative analysis. Despite the specimen consisting of 50 wt.% TiO_2_ nanomaterials (nano-TiO_2_) and 50 wt.% microscale ZnO powder, the nano-TiO_2_ quantitative result changes from 56.53% to 43.33% that occur as a variation of instrumental broadening are caused by divergence slit adjustment. This deviation could be accounted through a mathematical model that involves instrumental broadening. The research in this paper might provide a useful guide for developing an approach to measure accuracy quantification in unknown multi-phase mixtures

## 1. Introduction

Quantitative phase analysis (QPA), using X-ray diffraction (XRD), is considered of fundamental importance in technology used in the research of multi-phase mixtures [1]. Numerous approaches can be divided into two distinct groups: whole-pattern methods and single-peak methods [2]. Whole-pattern methods are performed by fitting a total range of patterns with parameters from crystal structure data [3,4,5], such as the Rietveld refinement method [6]. However, due to its complexity and time cost, this method is not suitable for use in geology, identification, or fabrications requiring rapid or large-volume sample quantification. In contrast, single-peak methods, using standards to calibrate the intensity ratio and then associating the measured intensity with the phase content in the specimen, are suitable for general application and are more accessible for establishing industry operating standards and enabling QPA in a number of rapid and convenient ways [7]. The reference intensity ratio (RIR) method [8] has been widely applied for the quantification of clay, minerals, medicines, etc. [9,10,11]. This application was promoted by the International Centre for Diffraction Data (ICDD), which applied α-Al_2_O_3_ as the standard calibration constants, known as I/I_corundum_ (I/I_c_, also known as the RIR value), to compare the diffraction intensities of powder specimens [12,13]. To meet the needs of users, the ICDD is exploring methods to add additional RIR values to the Powder Diffraction File (PDF) [12].

Fast and accurate quantification is essential for nanotechnology, which is one of the most dynamically developing fields of science and it is incredibly profitable from the manufacturers’ point of view [14,15,16,17,18]. The RIR method based on the fundamental intensity–concentration equation was commonly believed to be highly accurate, but the analysis results mostly depend on specimen and instrument factors [9,10,19]. Often, the mixture specimen is non-routine or totally unknown, typically containing nanocrystals, and there are various properties in each phase that manifest differences in the peak shape. Generally, nanocrystals create a decrease in the peak height and an increase in the peak’s full width at a half-maximum height (FWHM). The FWHM in the XRD pattern consists of two distinct parts: structure broadening (SB) due to the crystal structure and instrumental broadening (IB) controlled by optics, collimators, counters, etc. [20]. Meanwhile, FWHM broadening due to instruments has not attracted enough attention in quantitative deviation analysis.

In this study, an artificial mixture with a TiO_2_ nanomaterial (nano-TiO_2_) and microscale ZnO powder was prepared to obtain completely different SB in XRD patterns, and different IB were obtained by adjusting the divergence cross-slit, and the quantitative deviation of nano-TiO_2_ was investigated. We reported a quantitative deviation fluctuation with IB: a drop in the nano-TiO_2_ weight fraction (wt.%) from 56.53% to 43.33%, while the known nano-TiO_2_ wt.% is 50%. A mathematical model was built to quantitatively decipher the RIR method’s deviation. Research in this paper might provide a useful guide for developing an approach to measure accuracy quantification in unknown multi-phase mixtures.

## 2. Materials and Methods

**Figure 1:**
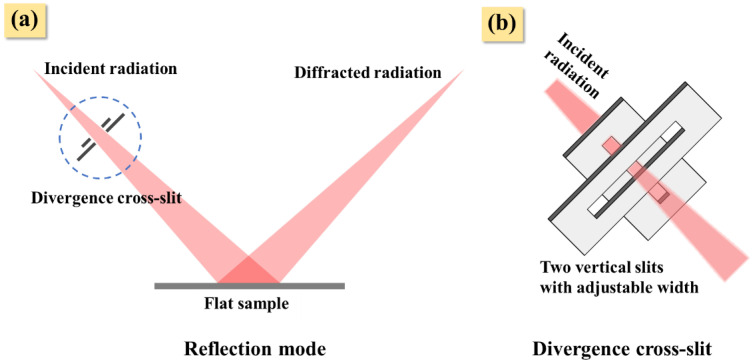
Schematic of diffraction experiment: in reflection mode, the incident radiation can be modified by changing the width of the divergence cross-slit (**a**). Divergent cross-slit: composed of two vertical variable slits, the slit width can be adjusted to change the passing area of incident radiation (**b**).

Nano-TiO_2_ powder with a particle size less than 100 nm and microscale ZnO powder with particle size of approximately 1 µm were obtained from commercial products and were artificially mixed according to the weight ratio designed by the XRD experiment. The IB was obtained by using a constant instrument parameter to measure silicon powder standard specimen. The mixtures were measured in reflection mode (Figure 1a), and the divergence cross-slits were installed on the incident radiation path (Figure 1b). The TiO_2_-ZnO (1:1) mixture was selected for XRD measurement under the designed divergent cross-slit width, as shown in Table 1.

Instrument parameters: Powder X-ray diffraction was performed using an intelligent diffractometer (Malvern-Panalytical Company, Almelo, The Netherlands), Pixcel 1D detector, BBHD-module filter, voltage 40 kV, current 40 mA, step length 0.02626°, dwell time per step of 127 s (total measurement time of 30 min per specimen). Observation of powder morphology was carried out using a VEGA 3SBH tungsten filament scanning electron microscope (TESCAN Inc., Brno, Czech Republic). 

Software: The quantitative analysis of XRD patterns was performed using Jade-standard 8.3 software, with pseudo-voigt function (PVF) for single peak fitting and total range fitting. The PVF is one of the peak shape functions to determine the peak shape parameters (FWHM, peak height, and integral area) [21]. Rietveld refinement was performed using Fullprof Suite January 2021 software [22], as shown in Figure S1.

## 3. Results

**Figure 2:**
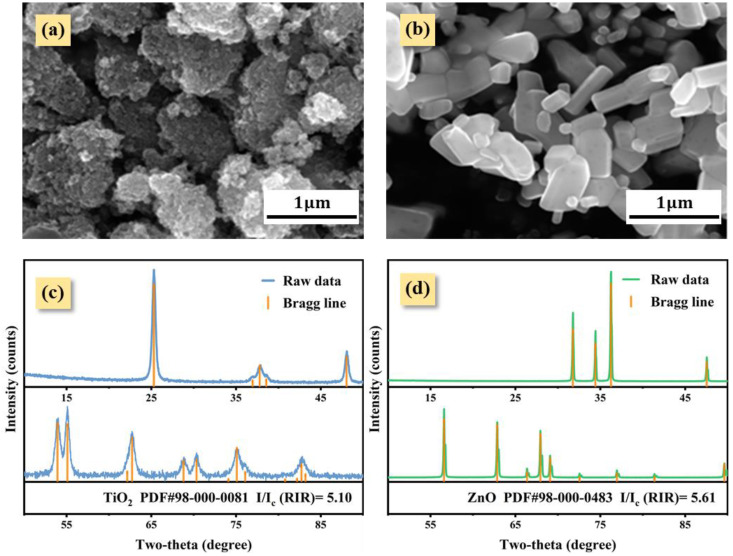
The SEM morphologies (**a**,**b**) and XRD patterns (**c**,**d**) of nano-TiO_2_ powder and ZnO powder. The information of PDF card and RIR value based on Jade-standard software were listed [12].

Figure 2 displays the morphologies and XRD patterns of nano-TiO_2_ and ZnO powder. The Nano-TiO_2_ powder exhibited agglomeration due to its ultra-fine particle size (Figure 1a), and the ZnO powder was in the shape of a smooth block (Figure 2b). Nano-TiO_2_ and ZnO, respectively, matched the Bragg lines in Powder Diffraction File (PDF) (Figure 2c,d), which is a case of an isotropic in crystal a structure and its related specimen preparation. Subsequently, the RIR values of nano-TiO_2_ and ZnO were chosen as 5.10 and 5.61.

**Figure 3:**
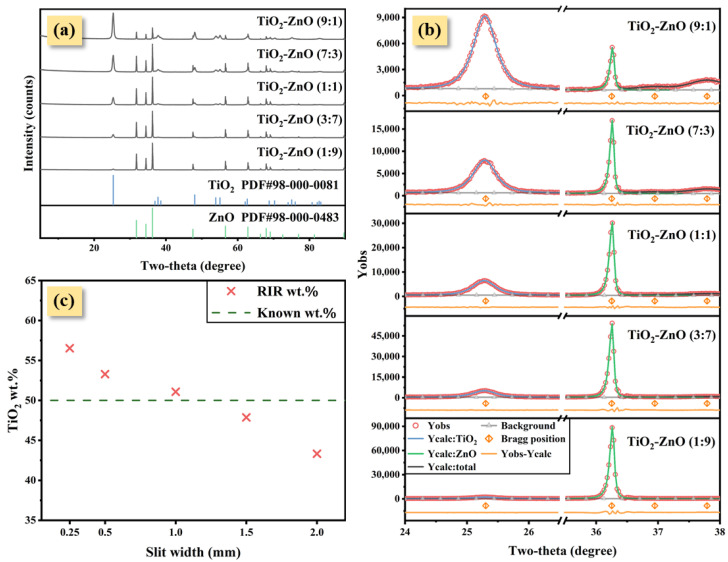
The XRD patterns of five artificial mixtures with varying weight fraction ratios (**a**). The strongest peaks of nano-TiO_2_ and ZnO were fitted using PVF to obtain peak shape parameters (**b**). Then, the IB curve obtained by fitting the FWHM of the five strongest peaks of the silicon powder standard was compared with the strongest peak FWHM of nano-TiO_2_ and ZnO (two-theta at 25.321°, 36.251°, respectively) (**c**).

Determining the SB of nano-TiO_2_ and ZnO before changing the IB by adjusting the cross-slit is necessary. The XRD patterns of different known wt.% were reported in Figure 3a. As the nano-TiO_2_ wt.% increases, the observed intensity of nano-TiO_2_ gradually increases, at the same time that the intensity of ZnO decreases. The single-peak fitting results of nano-TiO_2_ and ZnO (two-theta at 25.321°, 36.251°, respectively) are displayed in Figure 3b, and peak shape parameters are listed in Table 2. It is important to note that although the nano-TiO_2_ wt.% in different mixtures was changing, the FWHM of nano-TiO_2_ and ZnO remained constant due to their respective crystal structures (Figure 3c). The FWHM of nano-TiO_2_ (two-theta at 25.321°) is much larger than ZnO and the silicon powder standard, and the FWHM of ZnO is approximately equal to IB obtained from silicon powder standard. The average diameter of the nano-TiO_2_ crystal is 23.40 nm, obtained using the Scherrer formula:

(1) $$D_{\left(\mathrm{hkl}\right)}=\frac{K\lambda }{\mathrm{SB}\times \cos\theta }$$

where SB is the peak broadening derived from the measured FWHM corrected for the instrumental broadening (IB); K is the Scherrer constant, which usually takes a value of about 0.9 [23]; λ is the wavelength of the incident radiation; and θ is the diffraction angle.

**Figure 4:**
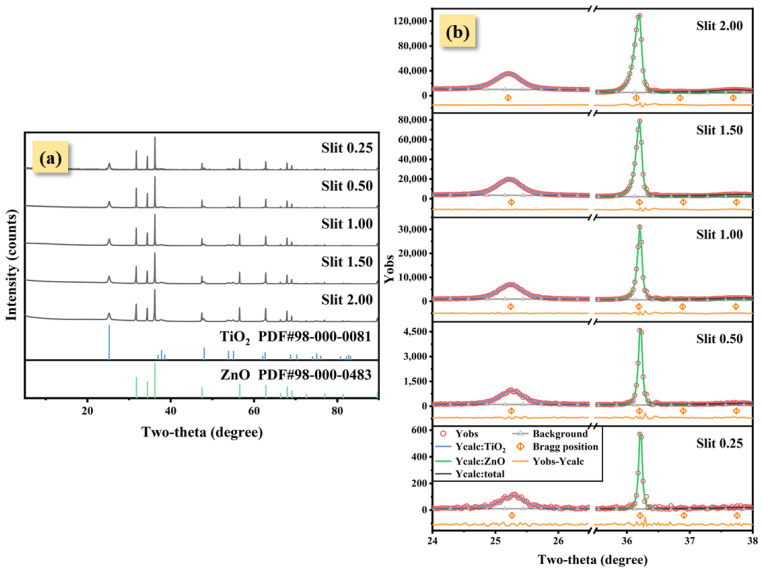
TiO_2_-ZnO (1:1)’s XRD patterns under five cross-slit widths (**a**). The strongest peaks of nano-TiO_2_ and ZnO were fitted using PVF to obtain peak shape parameters (**b**).

Five XRD experiments under different divergence cross-slit widths were performed using TiO_2_-ZnO (1:1) specimen (Figure 4a). With an increase in the cross-slit width, the FWHM of the strongest peaks of nano-TiO_2_ and ZnO both increased (Figure 4b). Intriguingly, as the cross-slit width increased, the RIR method wt.% of the nano-TiO_2_ decreased from 56.53% to 43.33% when the known nano-TiO_2_ wt.% was 50% (Table 3).

## 4. Discussion

**Figure 5:**
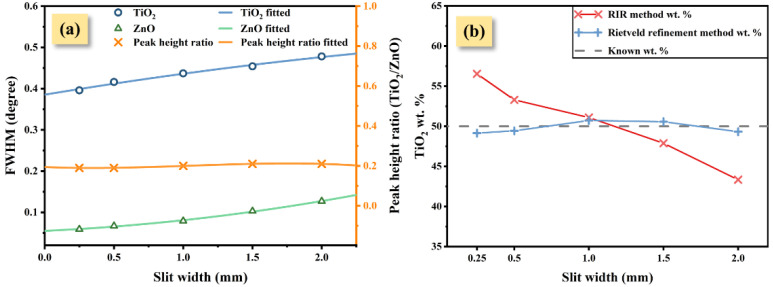
The change curve of the FWHM and height ratio of the strongest peaks (**a**) and quantitative result of RIR and Rietveld refinement method (**b**) under five slit widths.

The primary XRD quantitative principle should obey the fundamental equations relating diffraction intensity $\left(I_{i}\right)$ to concentration $\left(X_{i}\right)$ [24]. The RIR method with a powder specimen is as follows:

(2) $$X_{i}=\frac{I_{i}}{{\mathrm{RIR}}_{i}}\times {\left(\overset{n}{\underset{k=1}{\sum }}\frac{I_{k}}{{\mathrm{RIR}}_{k}}\right)}^{-1}$$

where $X_{i}$ is the wt.% of the phase in the mixture, $RIR_{i}$ is the RIR value [8], and $I_{i}$ is the diffraction intensities or integral area of the diffraction peak. The quantitative result can be directly calculated using the intensities (integral area) of the strongest peaks when the RIR value is determined, in which intensities are associated with FWHM and peak height. Figure 5a shows the FWHM-peak height change curve of nano-TiO_2_ and ZnO with different slit widths. Both FWHM of nano-TiO_2_ and ZnO increased as IB is increased (controlled by the divergent slit width), but the peak height ratio in each pattern is approximately constant at 0.2. When IB decreases, the intensity of ZnO falls faster than nano-TiO_2_ due to the fact that the FWHM of ZnO has an almost complete dependency on IB, and vice versa. Obviously, IB induced an unequal intensity contribution of the strongest peaks to nano-TiO_2_ and ZnO when nano-TiO_2_ provided SB in the mixture XRD pattern, which caused a quantitative deviation in the RIR method. However, such a trend of quantitative deviation in the RIR method does not appear when using the Rietveld refinement method.

**Figure 6:**
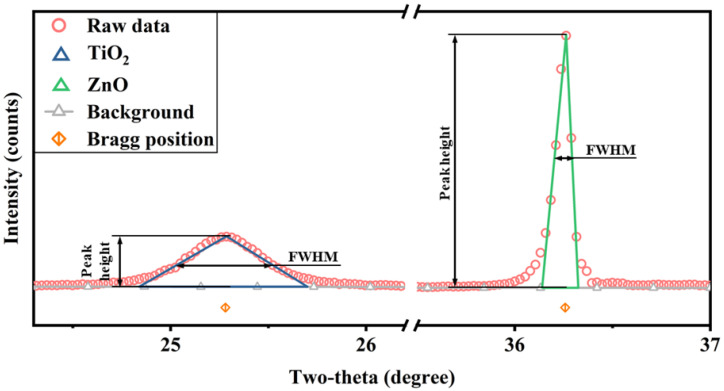
Approximate model of diffraction peak, in which the integral area is approximately FWHM multiplied by the peak height.

To decipher the significant quantitative deviation of the RIR method, IB must be extracted from FWHM in each phase. A mathematical model was built to establish a functional relation between IB and quantitative results and made the following assumptions:

(1) The existing phases α and β have a determined crystal structure, and the reference intensity ratio (RIR) of each phase can be obtained through an XRD experiment.
(2) We can use isosceles triangles to approximate the shape of the phase diffraction peaks (Figure 6). The integral area of the diffraction peak is approximately the product of the half-height of the isosceles triangle and its height; $I_{i}$ in Equation (2) can be elucidated as

(3) I=FWHM×H

where H is height of the strongest diffraction peak observed.

(3) There are powders α and β, where the FWHM of the diffraction peak of α is significantly increased due to the nanocrystal (larger than IB), and the FWHM of β is almost close to the instrumental broadening to the extent that they can be approximately equal.

The FWHM of the diffraction peaks of α and β is approximately:

(4) $${\mathrm{FWHM}}_{\alpha }\approx {\mathrm{SB}}_{\alpha }+\mathrm{IB},{\;\mathrm{FWHM}}_{\beta }\approx \mathrm{IB}$$

where ${\mathrm{SB}}_{\alpha }$ can be calculated from the Scherrer formula (Equation (1)). Combining the above assumptions, the calculation of a wt.% based on the RIR method can be written as

(5) $$W_{\alpha }=\frac{I_{\alpha }}{{\mathrm{RIR}}_{\alpha }}\times {\left(\frac{I_{\alpha }}{{\mathrm{RIR}}_{\alpha }}+\frac{I_{\beta }}{{\mathrm{RIR}}_{\beta }}\right)}^{-1}\approx \frac{\left({\mathrm{SB}}_{\alpha }+\mathrm{IB}\right)H_{\alpha }}{{\mathrm{RIR}}_{\alpha }}\times {\left(\frac{\left({\mathrm{SB}}_{\alpha }+\mathrm{IB}\right)H_{\alpha }}{{\mathrm{RIR}}_{\alpha }}+\frac{\left(\mathrm{IB}\right)H_{\beta }}{{\mathrm{RIR}}_{\beta }}\right)}^{-1}$$

When $H_{\beta }/H_{\alpha }$ are constant, make as

(6) $$H_{\beta }/H_{\alpha }=h,{\;\mathrm{RIR}}_{\beta }/{\mathrm{RIR}}_{\alpha }=r$$

where h is the peak height ratio and r is the RIR value ratio. Then, Equation (5) will be:

(7) $$W_{\alpha }\approx \frac{\left({\mathrm{SB}}_{\alpha }+\mathrm{IB}\right)\times H_{\alpha }}{{\mathrm{RIR}}_{\alpha }}\times {\left(\frac{\left({\mathrm{SB}}_{\alpha }+\mathrm{IB}\right)\times H_{\alpha }}{{\mathrm{RIR}}_{\alpha }}+\frac{\mathrm{IB}\times H_{\alpha }\times h}{{\mathrm{RIR}}_{\alpha }\times r}\right)}^{-1}=\frac{1}{1+h\times \mathrm{IB}\times {\left[\left({\mathrm{SB}}_{\alpha }+\mathrm{IB}\right)r\right]}^{-1}}$$

**Figure 7:**
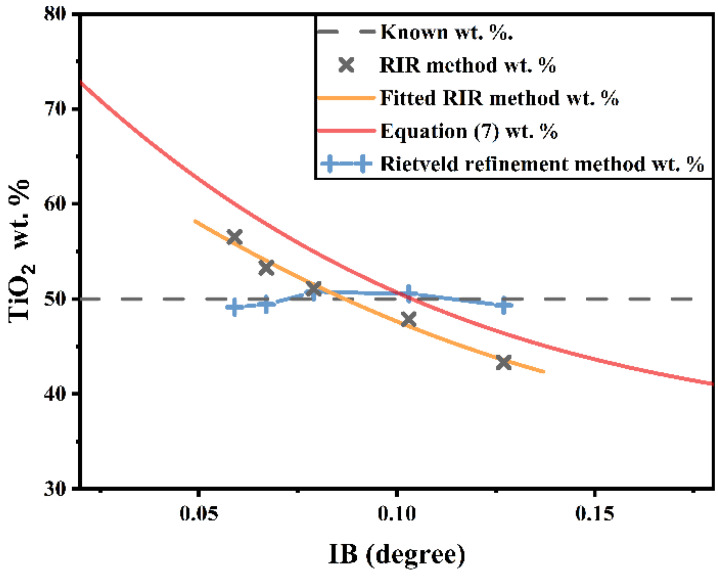
Nano-TiO_2_ quantitative curve based on Equation (7), which are compared to known wt.%, experimental wt.% and Rietveld refinement wt.%.

Then, we obtain the peak shape and RIR value from Table 3 to plot the wt.% curve of nano-TiO_2_ (Figure 7):

(8) $${\mathrm{SB}}_{\alpha }=0.354{}^{\circ},\;h=0.2\;,{\;\mathrm{RIR}}_{\alpha }=5.10,{\;\mathrm{RIR}}_{\beta }=5.61.$$

Figure 7 shows the wt.% comparison of the quantitative result of the known results, Equation (7), RIR, and Rietveld refinement methods. The curve of Equation (7)’s wt.% has the same tendency as the RIR’s wt.%. There is, however, a slight discrepancy. The isosceles triangle is not a perfect substitute for the diffraction peak, which should be the main reason for the discrepancy. It is also important to note that different quantitative deviation trends appeared in the RIR and Rietveld refinement methods. In the RIR method, the nano-TiO_2_ wt.% is under the influence of IB variation, whereas the nano-TiO_2_ wt.% is basically unchanged in the Rietveld refinement method (absolute deviation < 1%).

The quantitative deviation of RIR method can be explained by the simplification of Equation (7):

(9) $$W_{\alpha }\approx \frac{1}{1+h\times \mathrm{IB}\times {\left[\left({\mathrm{SB}}_{\alpha }+\mathrm{IB}\right)r\right]}^{-1}}$$

When SB is induced by a crystal structure in one or more phases in a mixture, $W_{\alpha }$ is strongly dependent on IB alteration, which is demonstrated by an inverse proportional relationship (Figure 7), and quantitative deviation is limited by the structure of Equation (7). We do not know the actual wt.% of each phase in a mixture when a specimen is obtained from a mountain or soil that contains nanocrystals, strain, etc., and so it can be hard to eliminate the quantitative deviation. 

When SB=0, the $W_{\alpha }$ in the binary mixture is given as:

(10) $$W_{\alpha }\approx \frac{1}{1+h\times r^{-1}}=\frac{I_{\alpha }}{{\mathrm{RIR}}_{\alpha }}\times {\left(\frac{I_{\alpha }}{{\mathrm{RIR}}_{\alpha }}+\frac{I_{\beta }}{{\mathrm{RIR}}_{\beta }}\right)}^{-1}$$

The IB does not have a role in the weight fraction calculation. 

In contrast, the quantitative result of the Rietveld refinement method does not involve the functional form of the RIR method, which avoids such a quantitative deviation.

The mathematical model was tested for a three-phase mixture containing Si nanocrystals. the nano-Si caused peak broadening in XRD pattern due to nanocrystals (Figure S2), which quantitative result calculated by RIR method (Figure S3) has the same trend as the mathematical model (Table S1).

## 5. Conclusions

To quantify a mixture containing nano-TiO_2_, the quantitative deviation of the RIR method showed a strong dependence on instrumental broadening. When the cross-slit width increases, the diffraction peaks of nano-TiO_2_ are affected by two broadening sources, the nanocrystal structure and instrumental broadening, which causes nano-TiO_2′_s quantitative result to decrease from 56.53% to 43.33%.

A mathematical model was developed to show the quantitative deviation’s trend. However, correcting for the RIR method’s quantitative deviation is a tough issue before the diffraction’s physical nature is explained. Instrumental broadening as an instrument factor varies across laboratories and equipment models.

The peculiarity of nanocrystals in XRD produces an independent source of peak broadening due to the crystal structure, which causes a significant quantitative deviation in the RIR method. In addition, when the mixture contains nanocrystals, the Rietveld refinement method is recommended for quantitative phase analysis rather than the RIR method. The research in this paper might provide a useful guide for measuring accuracy quantification in mixtures containing nanocrystals.

## Figures and Tables

**Table 1:** TiO_2_-ZnO weight ratio and divergent cross-slit width of XRD experiment.

Weight Ratio, Artificial Mixtures of TiO_2_ and ZnO				
9:1	7:3	1:1	3:7	1:9
**Cross-slit width (mm), TiO_2_-ZnO (1:1)**				
0.25	0.50	1.00	1.50	2.00

**Table 2:** Peak shape parameters and quantitative results in five artificial mixtures.

Nano-TiO_2_, Known wt.%	FWHM of Nano-TiO_2_, 2θ at 25.321 (°)	FWHM of ZnO, 2θ at 25.321 (°)
90	0.429	0.088
70	0.432	0.089
50	0.430	0.089
30	0.430	0.086
10	0.434	0.082
Silicon powder standard	0.083	0.081

**Table 3:** TiO_2_-ZnO (1:1)’s peak shape parameters and quantitative results of RIR method under different cross-slit widths.

Slit Width (mm)	FWHM of TiO_2_ (°)	FWHM of ZnO (°)	Peak Height Ratio: TiO_2_-ZnO	RIR Method		Rietveld Refinement Method	
				wt.%	Deviation ^1^ (%)	wt.%	Deviation ^1^ (%)
0.25	0.396	0.059	0.19	56.53	+6.53	49.14	−0.86
0.50	0.416	0.067	0.19	53.29	+3.29	49.43	−0.57
1.00	0.437	0.079	0.20	51.09	+1.09	50.72	+0.72
1.50	0.454	0.103	0.21	47.88	−2.12	50.57	+0.57
2.00	0.478	0.127	0.21	43.33	−6.67	49.32	−0.68

^1^ Deviation (%) calculation: known weight fraction minus calculated result, where the known TiO_2_ wt.% is 50%.

## Data Availability

Not applicable.

## Supplementary Materials

The following supporting information can be downloaded at: https://www.mdpi.com/article/10.3390/nano12142320/s1, Figure S1: Quantitative analysis of five cross slit widths in Rietveld refinement method; Figure S2: XRD pattern of three-phase mixture of Si, ZnO and CaCO_3_; Figure S3: Fitting results of peak shape function under five slit widths. Diffraction peaks from left to right are the strongest peaks of nano-Si, CaCO_3_, ZnO; Table S1: Integral area of each phase and quantitative result of RIR method.
