# Supplementary material for: Quantitative Deviation of Nanocrystals Using the RIR Method in X-ray Diffraction (XRD)
Source: Nanomaterials (Basel). 2022 Jul 6;12(14):2320. doi: 10.3390/nano12142320 (PMC9325253; doi:10.3390/nano12142320)
Supplement: Supplementary file 1 [file nanomaterials-12-02320-s001.zip › nanomaterials-1753782-supplementary.pdf]

nanomaterials

*Supplementary Materials for*

# Quantitative deviation of nanocrystals using the RIR method in X-ray diffraction (XRD)

**Qinyuan Huang [3,4], Chunjian Wang [1,2]* and Quan Shan [3,4]****

## This PDF file includes:

Materials and Methods: Quantitative analysis of five cross slit widths in Rietveld refinement method.

Discussion: The mathematical model was tested for a three-phase mixture containing Si nano-crystals.

### 1. Materials and Methods

Software: The quantitative analysis of XRD patterns was performed using Jade-standard software, with pseudo-voigt function (PVF) for single peak fitting and total range fitting. The PVF is one of the peak shape functions to determine the peak shape parameters (FWHM, peak height and integral area) [1]. Rietveld refinement was performed using Fullprof software (**Figure S1**).

The software is suitable for the Rietveld Refinement method, the website is as follows https://www.ill.eu/sites/fullprof/index.html

The FullProf Suite (for Windows, Linux and macOS) is formed by a set of crystallographic programs mainly developed for Rietveld analysis (structure profile refinement) of neutron (constant wavelength, time of flight, nuclear and magnetic scattering) or X-ray powder diffraction data collected at constant or variable step in scattering angle 2theta [2].

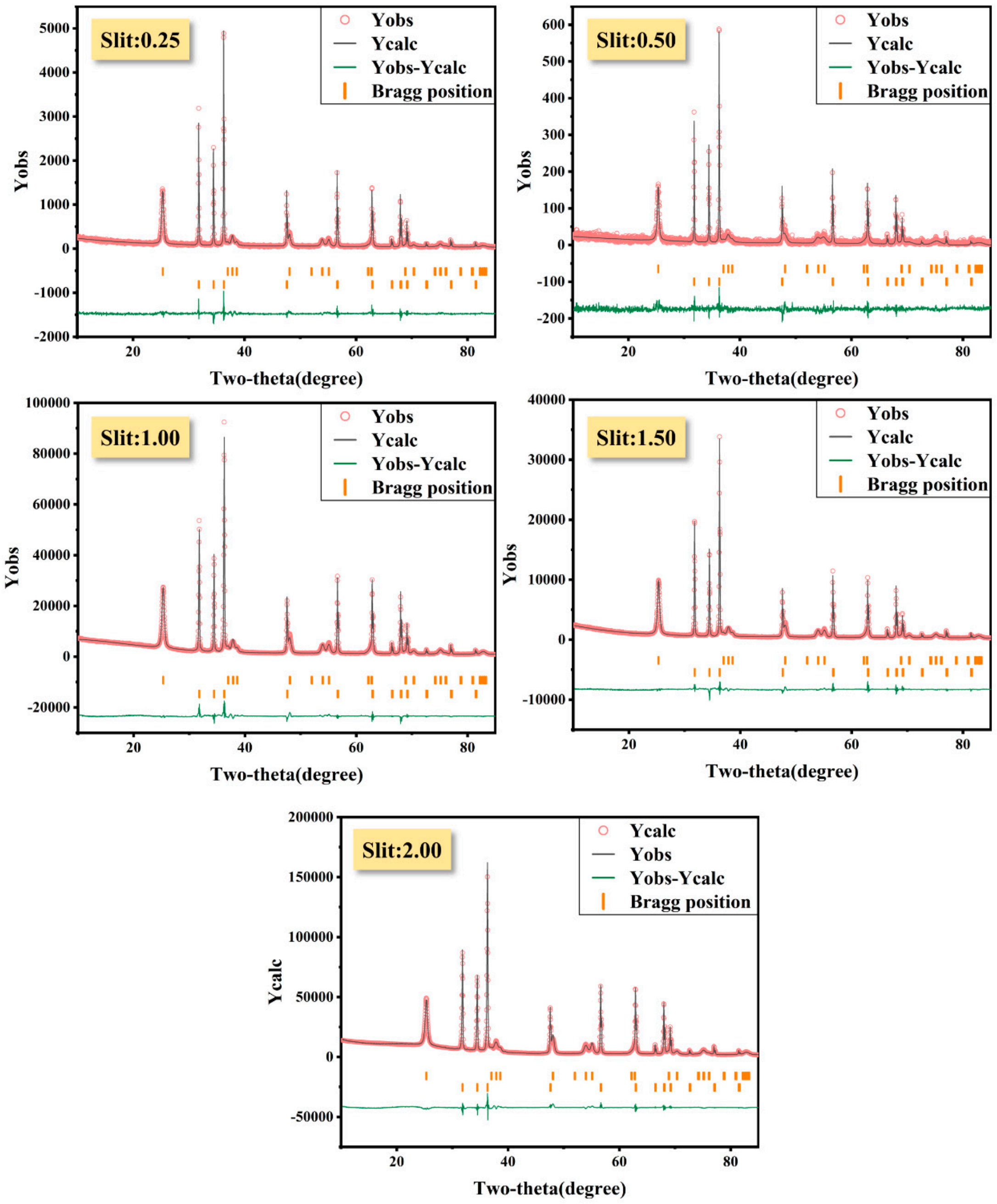


**Figure S1.** Quantitative analysis of five cross slit widths in Rietveld refinement method. Where the Yobs is observed intensity(counts), Ycalc is calculated curve, which are all represented on the same coordinate scale. The quantitative analysis was using PVF based on the Jade Standard software, and diffraction data of Cu K$\alpha$2 in experimental patterns was subtracted before the quantitative analysis.

## 2. Discussion

The mathematical model was tested for a three-phase mixture containing Si nanocrystals.

Nano-Si powder with particle size less than 100 nm and microscale ZnO powder, $CaCO_3$ powder with particle size approximately to 1-3 μm were obtained from commercial products, and were artificially mixed. Samples were mixed for 6 hours to ensure homogeneity. Interestingly, we did not count the weight fraction of each phase before mixing. XRD experiments were carried out on the same sample with divergent cross-slits of

different widths (**Figure S2**). All raw data were fitted by the pseudo-voigt functions to obtain the intensity and shape parameters of the diffraction peaks (**Figure S3**). Intensities of each diffraction peak were used for weight fraction calculation based on the RIR method (**Table S1**).

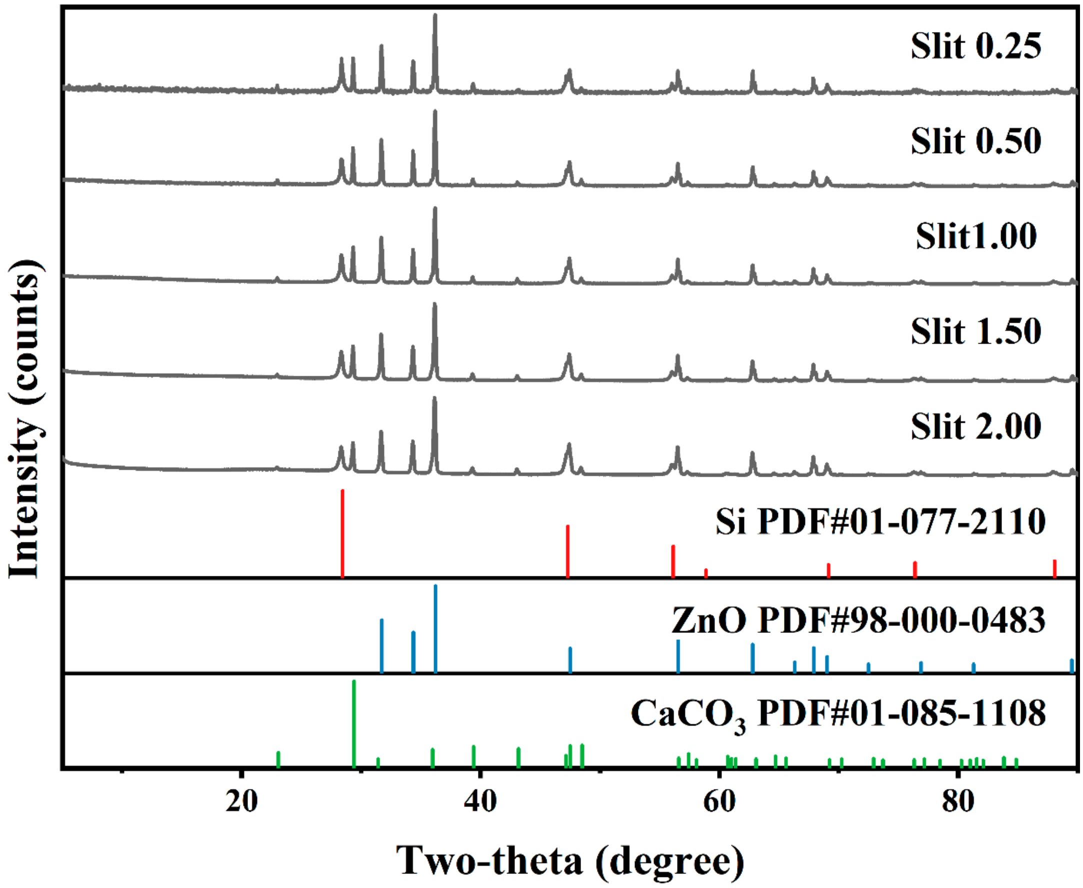


**Figure S2.** XRD pattern of three-phase mixture of Si, ZnO and $CaCO_3$.

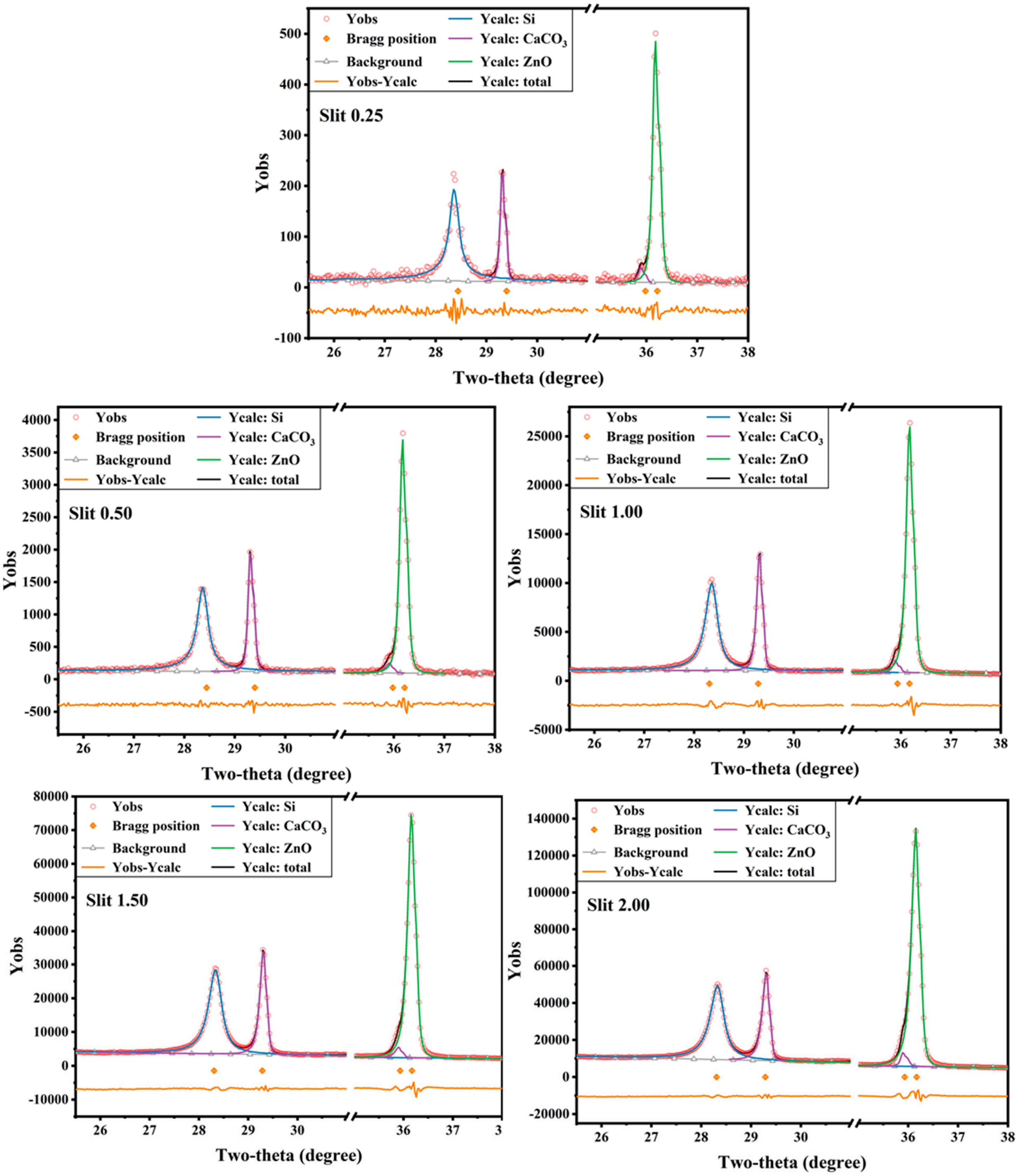


**Figure S3.** Fitting results of peak shape function under five slit widths. Diffraction peaks from left to right are the strongest peaks of nano-Si. $CaCO_3$, ZnO.

**Table S1.** Integral area of each phase and quantitative result of RIR method.

| Slit width (mm) | Integral area | | | Nano-Si |
|---|---|---|---|---|
| | Nano-Si | ZnO | $CaCO_3$ | wt.% |
| 0.25 | 1959 | 712 | 2236 | 41.02 |
| 0.50 | 16643 | 6873 | 17680 | 40.93 |
| 1.00 | 114505 | 49695 | 132930 | 39.17 |
| 1.50 | 329498 | 145571 | 418195 | 37.71 |
| 2.00 | 505119 | 253508 | 757978 | 34.20 |

**Footnote:** The RIR value of each phase was obtained from Powder Diffraction File (PDF): Si: 4.55, ZnO: 5.61, $CaCO_3$: 3.23.

The nano-Si caused peak broadening in XRD pattern due to nanocrystals. Nano-Si's wt.% calculated using the RIR method changes from 41.02 to 34.20 with the slit width increases, which has the same trend as the mathematical model. When the grain size and weight fraction of Si nanocrystals are unknown in the mixture, the actual content cannot be obtained using the RIR method. The research in this paper might provide a useful guide for developing an approach to measure accuracy quantification in unknown multi-phase mixtures.

## References

1. Alqahtani, H. A study of Asphalt Binders by X-Ray Diffraction Using Pearson-VII, Pseudo-Voigt and Generalized Fermi Functions. Memorial University of Newfoundland, 2017.
2. Rodriguez-Carvajal, J. Recent developments of the program FULLPROF, commission on powder diffraction. *IUCr Newsl.* **2001**, *26*.
